# Distinct amyloid-β and tau-associated microglia profiles in Alzheimer’s disease

**DOI:** 10.1007/s00401-021-02263-w

**Published:** 2021-02-20

**Authors:** Emma Gerrits, Nieske Brouwer, Susanne M. Kooistra, Maya E. Woodbury, Yannick Vermeiren, Mirjam Lambourne, Jan Mulder, Markus Kummer, Thomas Möller, Knut Biber, Wilfred F. A. den Dunnen, Peter P. De Deyn, Bart J. L. Eggen, Erik W. G. M. Boddeke

**Affiliations:** 1 Department of Biomedical Sciences of Cells and Systems, Section Molecular Neurobiology, University of Groningen and University Medical Center Groningen (UMCG), Antonius Deusinglaan 1, 9713AV Groningen, the Netherlands; 2 Foundational Neuroscience Center, AbbVie Inc, Cambridge, MA USA; 3 Department of Biomedical Sciences, Laboratory of Neurochemistry and Behavior, Institute Born-Bunge, University of Antwerp, Wilrijk, Antwerp, Belgium; 4 Department of Neurology and Alzheimer Center, University of Groningen and University Medical Center Groningen (UMCG), Groningen, the Netherlands; 5 Faculty of Medicine & Health Sciences, Translational Neurosciences, University of Antwerp, Antwerp, Belgium; 6 Division of Human Nutrition and Health, Chair group of Nutritional Biology, Wageningen University & Research, Wageningen, the Netherlands; 7 Department of Neuroscience, Karolinska Institute, Stockholm, Sweden; 8 Neuroscience Discovery, AbbVie Deutschland GmbH & Co. KG, Ludwigshafen, Germany; 9 Department of Pathology and Medical Biology, University Medical Center Groningen (UMCG), University of Groningen, Groningen, the Netherlands; 10 Department of Neurology, Memory Clinic of Hospital Network Antwerp (ZNA), Middelheim and Hoge Beuken, Antwerp, Belgium; 11 Center for Healthy Ageing, Department of Cellular and Molecular Medicine, University of Copenhagen, Blegdamsvej 3B, 2200 Copenhagen N, Denmark

**Keywords:** Microglia, Alzheimer’s disease, Single-nucleus RNA sequencing, Amyloid-β, Tau

## Abstract

Alzheimer’s disease (AD) is the most prevalent form of dementia and is characterized by abnormal extracellular aggregates of amyloid-β and intraneuronal hyperphosphorylated tau tangles and neuropil threads. Microglia, the tissue-resident macrophages of the central nervous system (CNS), are important for CNS homeostasis and implicated in AD pathology. In amyloid mouse models, a phagocytic/activated microglia phenotype has been identified. How increasing levels of amyloid-β and tau pathology affect human microglia transcriptional profiles is unknown. Here, we performed snRNAseq on 482,472 nuclei from non-demented control brains and AD brains containing only amyloid-β plaques or both amyloid-β plaques and tau pathology. Within the microglia population, distinct expression profiles were identified of which two were AD pathology-associated. The phagocytic/activated AD1-microglia population abundance strongly correlated with tissue amyloid-β load and localized to amyloid-β plaques. The AD2-microglia abundance strongly correlated with tissue phospho-tau load and these microglia were more abundant in samples with overt tau pathology. This full characterization of human disease-associated microglia phenotypes provides new insights in the pathophysiological role of microglia in AD and offers new targets for microglia-state-specific therapeutic strategies.

**Supplementary Information:**

The online version contains supplementary material available at 10.1007/s00401-021-02263-w.

## Introduction

Alzheimer’s disease (AD) is the most prevalent cause of dementia, affecting about 35 million people worldwide. AD is neuropathologically characterized by abnormal aggregation of extracellular amyloid-β and hyperphosphorylation of neuronal tau. These pathological abnormalities exert stress on various cell types in the brain, including neurons, oligodendrocytes, astrocytes, microglia, and vascular cells [31]. Microglia, the tissue-resident macrophages of the central nervous system (CNS) [21], are important for CNS homeostasis and are implicated in AD pathology and single-cell profiling of human microglia has first been reported by Masuda et al. (2019). In amyloid mouse models of AD, a phagocytic/activated microglia phenotype was identified (known as DAM/ARM/MGnD) [8, 18, 30]. It is unclear whether a similar microglia phenotype is present in the human brain. Recently, three single-cell transcriptomics studies based on human tissue indicated that neurons, oligodendrocytes, astrocytes and microglia are affected by AD pathology [11, 22, 26]. However, in these studies, changes associated with disease progression and transcriptional affects linked to amyloid-β and phospho-tau pathology on cellular transcriptional profiles were not reported.

The complex morphology of brain cells and the low availability of ‘fresh’ material (biopsies/necropsies) complicate single-cell RNA sequencing (scRNAseq) of human brain tissue. As an alternative, brain banks contain high numbers of frozen brain tissue samples, from which transcriptomic data can be generated. From frozen tissue, single nucleus can be analyzed as a reliable proxy for the cellular transcriptome [10]. Multiple frozen samples can be processed simultaneously allowing for balanced experimental designs, minimizing technical variation between experimental groups. In addition, frozen tissue samples allow for neuropathological examination prior to sample preparation, which does not always confirm the clinical diagnosis [1].

In the current study, amyloid-β and tau-pathology-associated transcriptional changes in AD were investigated through single-nucleus RNA sequencing (snRNAseq). snRNAseq has previously been used to successfully characterize human brain tissue from donors with AD, multiple sclerosis and autism spectrum disorder [11, 22, 29, 34]. In these studies, unsorted nuclei were profiled, resulting in datasets largely composed of neurons and oligodendrocytes and with relatively low numbers of microglia and other less abundant cell types. To overcome this limitation, we improved the isolation of nuclei of less abundant cell types from the far more numerous neuronal and oligodendrocyte nuclei in the total CNS pool, increasing the statistical power to detect disease-induced transcriptomic changes and progressive cell-state shifts in microglia and astrocytes.

## Materials and methods

### Human brain tissue and neuropathology

Brain tissue for snRNAseq was obtained from the NeuroBiobank of the Institute Born-Bunge (NBB-IBB), Wilrijk (Antwerp), Belgium (ID: BB190113) and donors gave informed consent to donate their brain to the NBB-IBB. Ethical approval was granted by the medical ethics committee of the Hospital Network Antwerp (ZNA, approval numbers 2805 and 2806). The study was compliant with the World Medical Association Declaration of Helsinki on Ethical Principles for Medical Research Involving Human Subjects.

Neuropathological evaluation of the brain was performed on the formalin-fixated right hemisphere. A standard selection of 10–13 regionally dissected brain regions, including frontal, temporal and occipital lobes (at the level of Brodmann area 17, area striata) of the neocortex, amygdala, hippocampus (at the level of the posterior part of the amygdala and the lateral geniculate body), basal ganglia, thalamus, brainstem, substantia nigra, pons at the level of the locus coeruleus and cerebellum (including dentate gyrus), was embedded in paraffin and routinely stained with hematoxylin and eosin, cresyl violet and Bodian’s method, allowing neuropathological confirmation or rejection of the clinically-established diagnosis. Furthermore, routine examination of immunoreactivity against amyloid-β (clone 4G8) and P-tau181-P (clone AT8) was performed, as well as detection of hyperphosphorylated TAR DNA-binding protein-43 (TDP)-43 and ubiquitin. When the presence of Lewy bodies was suspected based on the hematoxylin and eosin and ubiquitin immunoreactivity, an anti-α-synuclein staining was included to rule out Parkinson’s disease.

The staining procedures of the NBB-IBB are standardized and as follows: paraffin-embedded tissue sections were de-paraffinized in xylene followed by an ethanol series from 100 to 70% and rinsed in tap water. Antigen retrieval was performed for the amyloid-β staining by incubation in 80% formic acid for 5 min. For the phospho-tau staining, no antigen retrieval was performed. Peroxidase blocking was performed with 1% H_2_O_2_ in methanol for 30 min. The sections were put in TBS (pH 7.4) and blocked with normal goat serum (1:25) in 1% BSA/TBS. Then, the sections were incubated with primary antibody in 1% BSA/TBS overnight (AT8 1:10,000 for tau, own production; 4G8 1:10,000 for amyloid-β, Senetek). Slides were then washed in TBS and incubated for 30 min with secondary antibody (goat-anti-mouse IgG, 1:500) in 1% BSA/TBS. After washing with TBS, the sections were incubated for 30 min with Avidin–Biotin Complex. The sections were incubated in a DAB solution (0.05% in TBS with six drops NaOH and 12.5 μL of H_2_O_2_). Sections were rinsed in water and counterstained with Hematoxylin for 1 min. The sections were dehydrated in graded ethanol (70–100%) followed by xylene. Lastly, coverslips were mounted with HistoRAL.

AD patients were neuropathologically diagnosed according to the criteria of [5, 6] and [15]. Alternatively, on samples collected after May 2011, the ABC-scoring method of [23] to assess low, intermediate or high AD neuropathologic change, was applied to AD brains. Ten donors that met the AD-criteria, based on one of the above-mentioned strategies, were used in the snRNAseq experiment and eight donors without neurological disease (CTR). For each donor, two brain regions were included: the occipital cortex (OC) and the occipitotemporal cortex (OTC; fusiform gyrus). In the samples of the AD donors, the OC contained amyloid-β pathology and no or low-level tau-pathology; the OTC contained both amyloid-β pathology and tau-pathology. Five of the CTR donors were clean of amyloid-β or tau-pathology in all the analyzed regions; these are referred to as CTR. Three CTR donors did show some low-degree amyloid pathology in the OC and OTC regions, these are referred to as CTR + . To reduce regional variation between samples, we isolated nuclei from grey-matter areas only.

For immunofluorescent P2RY12/IBA1/ITGAX staining in Fig. 3, formalin-fixed and paraffin-embedded frontal cortex samples of donors with Alzheimers disease (*n* = 10), dementia with Lewy bodies (*n* = 10) and age-matched controls (*n* = 9) were provided by the Netherlands brain bank. Tissue microarray (TMA) containing 2 cores (grey matter; diameter 1 mm) of each donor [17] was cut into 5 µm thick slices and collected on a superfrost + microscope slide.

For immunofluorescent GRID2/IBA1/TAU staining in Fig. 4, formalin-fixed, paraffin-embedded brain tissue from six CTR and ten AD donors of the hippocampus with entorhinal allocortex and temporal isocortex were derived from autopsies performed in the north-east of the Netherlands according to the “Code Goed Gebruik Patientenmateriaal”.

### Tissue selection based on RNA quality

About 50 mg of tissue was used for RNA isolation with the RNeasy Lipid Tissue Mini Kit from Qiagen, according to the manufacturer’s protocol. Quality of the RNA was determined using the Experion™ Automated Electrophoresis system (BIO-RAD) and only samples with a RIN > 5 were included in the experiment.

### Nuclei isolation

**Fig. 1:**
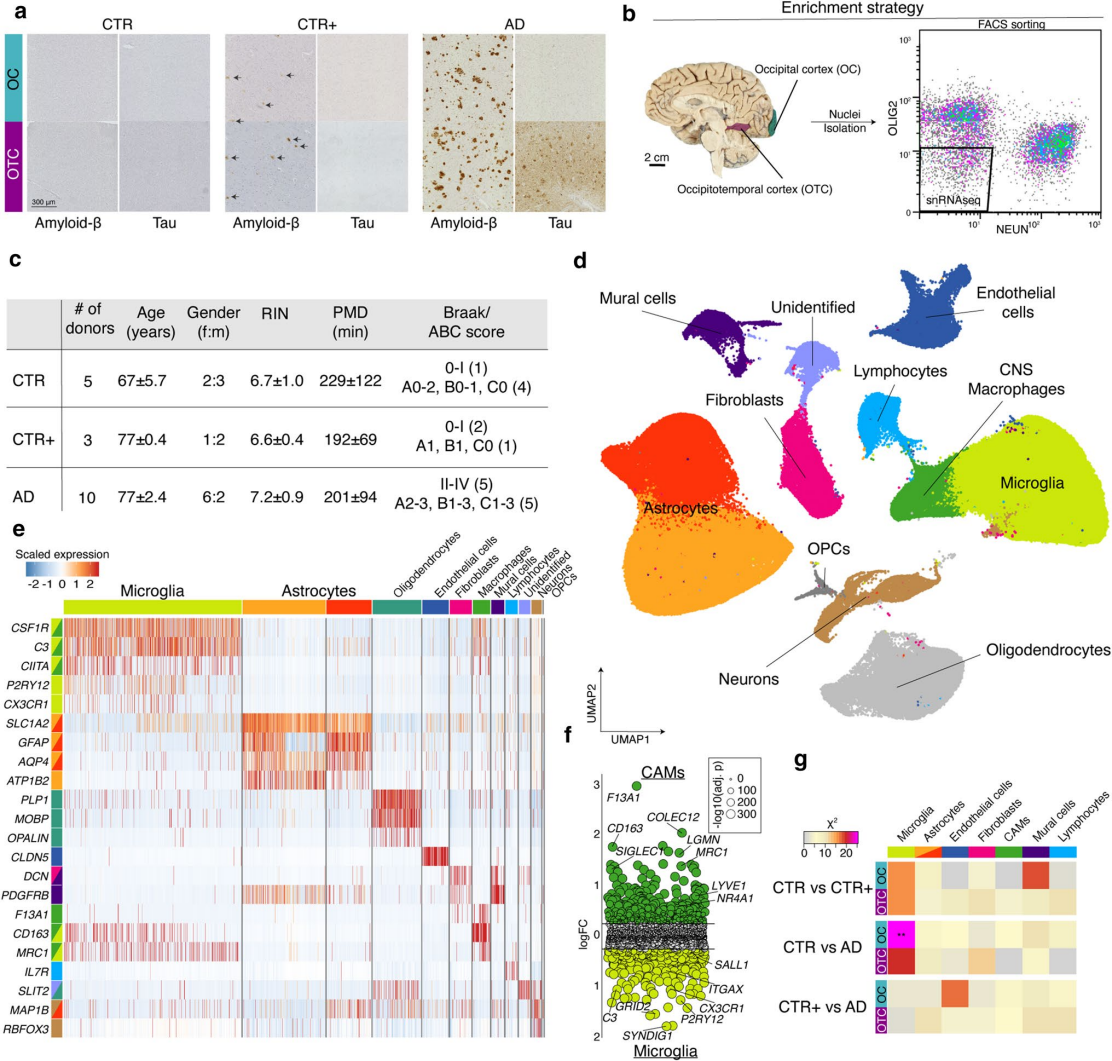
Enrichment yields high numbers of microglia and astrocytes for snRNAseq**.** **a** Pathological hallmarks of donor groups. **b** Enrichment strategy for NEUN^neg^ and OLIG2^neg^ nuclei. (Brain Image courtesy of the Neurobiobank of the Institute Born-Bunge, Antwerp (Wilrijk), Belgium (NB190113)). **c** Donor information. Age, RIN and PMD are presented as mean ± SD. **d** UMAP depicting 482,472 nuclei derived from 36 human cortical brain samples. Colors indicate cell type clusters. **e** Heatmap depicting expression of selected cell type marker genes. **f** Dot plot depicting logFC per gene from the comparison CAM versus microglia nuclei. Size depicts significance level. **g** Heatmap depicting Chi-squared associations between subcluster distribution within each cell type and donor group per brain region. **: *p* < 0.01. *OC* Occipital Cortex; *OTC* Occipitotemporal Cortex; *RIN* RNA integrity number; *PMD* Postmortem delay; *CTR* non-demented controls; *CTR+ * non-demented controls with mild amyloid-β pathology; *AD* clinical and neuropathological Alzheimer’s disease

Fresh frozen brain tissue of the left hemisphere was used for snRNAseq. Nuclei isolation and sorting were performed on multiple days with two donors (one CTR and one AD) per day, with both regions of the same donor in the same batch. Nuclei were isolated as described in van [4] with a few adaptations (Fig. 1a). In brief, from each tissue block, 30–40 cryostat sections of 40 µm were cut and collected and lysed in a sucrose lysis buffer (10 mM Tris–HCL (pH 8.0); 320 mM sucrose; 5 mM CaCl_2_; 3 μM Mg(Ac)_2_; 0.1 mM EDTA; 1 mM dithiothreitol (DTT) and 0.1% Triton X-100). The lysates were filtered through a 70 µm cell strainer. Nuclei were purified by ultracentrifugation (107,000× *g* for 1.5 h at 4 °C) through a dense sucrose buffer (10 mM Tris–HCL (pH 8.0); 1.8 M sucrose; 3 μM Mg(Ac)_2_; 0.1 mM EDTA and 1 mM DTT). The supernatants were removed and pellets were re-suspended in 2% BSA/PBS containing RNase inhibitor (0.35 U/μL) (Thermo Fisher Scientific). Samples were kept on ice throughout the isolation and staining procedure. The nuclei were incubated with fluorescently-conjugated antibodies directed against the neuronal marker NEUN (RBFOX3/NEUN (1B7) AF647 mouse mAB, Novus Biologicals, NBP1-92693AF647) and the transcription factor OLIG2 for the oligodendrocyte lineage (Anti-OLIG2 clone 211F1.1 AF488 mouse mAb, Merck Millipore, MABN50A4). After washing, the DNA dye DAPI was added and nuclei were sorted on a MoFlo Astrios. For each sample, we collected DAPI^pos^NEUN^neg^OLIG2^neg^ nuclei for snRNAseq and DAPI^pos^NEUN^pos^OLIG2^neg^ and DAPI^pos^NEUN^neg^OLIG2^pos^ for bulk RNAseq (Figure S1).

### Bulk RNAseq library construction and sequencing

RNA was isolated from nuclei pellets with the Arcturus™ PicoPure™ RNA Isolation Kit from Thermo Fisher Scientific. RNA concentrations were measured on a Qubit using a HS RNA kit. 3 ng of the DAPI^pos^NEUN^neg^OLIG2^pos^ samples and 8 ng of the DAPI^pos^NEUN^pos^OLIG2^neg^ samples was used for library preparation with the Lexogen QuantSeq 3′ mRNA-Seq Library Prep Kit (FWD) from Illumina. All libraries were pooled equimolarly and sequenced on a NextSeq 500 at the sequencing facility in the UMCG.

### snRNAseq library construction and sequencing

The single-nucleus cDNA libraries were constructed using the Chromium Single Cell 3′ Reagents Kit v3 and corresponding user guide (10 × Genomics). All samples were pooled in equimolar ratios and sequenced on a NextSeq 500 (v2.5) at GenomeScan in Leiden and the sequencing facility in the UMCG.

### Immunohistochemistry and imaging

Paraffin blocks from four donors were cut at 5 μm thickness and sections were placed on Superfrost plus glass slides. Sections were de-paraffinized, rehydrated and subjected to a heat-induced epitope retrieval by cooking in 10 mM Sodium Citrate with 0.05% Tween (pH 6.0) for 10 min. Endogenous peroxidase was blocked with H_2_O_2_ (0.3%) in PBS for 30 min. Sections were washed and blocked with 2% normal donkey serum and 2% bovine serum albumin. Sections were incubated overnight with primary antibody (rabbit-anti-GRID2, Abcam ab251953, 1:100), washed with PBS and incubated with a biotinylated donkey-anti-rabbit secondary antibody (Vector BA-1000, 1:400). For visualization, the sections were incubated with Avidin–Biotin Complex (Vector PK-6100) treated with DAB with 0.03% H_2_O_2_ in PBS. Counterstaining was performed with cresyl violet. Slides were dehydrated in a graded ethanol series and coverslips were mounted with DePex. Sections were imaged with a Hamamatsu Nanozoomer.

### Immunofluorescence and imaging

For the immunofluorescent staining in Fig. 3, the protocol described by Weidner et al. [35] with minor modifications was used. Briefly, slides were pre-treated in a BOND-RX automated stainer (Leica Biosystems, Wetzlar, Germany). First the sections were”baked” (30 min at 60 °C) and dewaxed using Bond Dewax Solution (Leica Biosystems, 72 °C). For the epitope retrieval, a heat-induced epitope retrieval step (Citrate-based solution, pH 6.0, 20 min at 100 °C) was followed by endogenous peroxidase block (0.03% H_2_O_2_). To detect low abundant targets and minimize off-target binding, primary antibodies (Rabbit-anti-ITGAX, proteinatlas.org, 1:200; Rabbit-anti-P2RY12, proteinatlas.org, 1:100; Goat-anti-IBA1, Abcam, 1:300; 0.1 M phosphate buffer, pH 7.4, containing 0.3% TX-100 and 0.1% NaN_3_) were incubated overnight at 4 °C. After blocking in Tris-buffered saline containing 2.5% blocking reagent, the secondary antibodies diluted in blocking buffer or polymer (Polymer-HRP, ThermoFisher, Donkey-anti-Goat-HRP, Jackson, 1:300) were applied to the slides. Antibody binding was visualized using tyramide signal amplification (TSA; 1:150; Akoya) using Fluorescein, cy3.5 and cy5 conjugated tyramide. For sequential multiplex TSA experiments, secondary antibody-conjugated peroxidase was blocked by applying NaN_3_ between staining rounds. For experiments using multiple antibodies raised in the same species, an additional epitope retrieval step (Citrate-based, pH 6.0, 20 min at 100 °C) between staining rounds was used to elute antibodies from the tissue without affecting tyramide binding. Lipofuscin auto-fluorescence was blocked by Sudan Black B (1% in 70% ethanol; Sigma-Aldrich, St. Louis, MO, USA) before mounting (ProLong Gold Antifade Mountant with DAPI). Images of the slides were acquired on an automated VSlide slide scanning system (Metasystems, Altlussheim, Germany). TMA slides were imaged with a 20 × objective. Each field of view was captured at 3 z‐levels with a 1 μm interval to create an extended focus image. Acquired fields of view images were stitched to create a complete overview with microscopic resolution. The emission spectra for the fluorophore‐conjugated secondary antibodies were as follows: Hoechst (420‐485 nm), fluorescein (494–517) Cy3.5 (580–595 nm), and Cy5 (650–670 nm). High-resolution images of individual cells were captured using a laser confocal microscope (LSM880, Zeiss) optimized for best separation of fluorescence signals.

For immunofluorescent staining in Figure 4, sections were de-paraffinized and rehydrated and subjected to a heat-induced epitope retrieval procedure for 10 min using a Sodium Citrate buffer pH 6.0 plus 0.05% Tween. After cooling down for 30 min, sections were incubated for 30 min in 0.1% Sodium borohydride in PBS followed by a 5-min incubation in 0.5% Sudan Black B (Sigma S0395) in 70% ethanol. Both incubations were included to reduce auto-fluorescence. After three washes in distilled water and PBS, sections were pre-incubated with 2% normal donkey serum and 2% bovine serum albumin in PBS for 30 min, before incubation with a mixture of primary antibodies. These mixtures contained an antibody raised in goat (IBA1, Abcam ab5076, 1:500), mouse (1:200; Phospho-TAU, Thermo Fisher Scientific MN1020, 1:500; SPP1, DSHB MPIIIB10(1), 1:100) and rabbit (GRID2, Abcam ab251953, 1:100) in PBS with 2% donkey serum. After an overnight incubation, sections were washed with PBS and subsequently incubated with a sections were washed with PBS and incubated for 2 h with a biotin-conjugated donkey anti-rabbit IgG (Jackson Immuno Research 711-065-152) followed by a PBS wash and a 1 h incubation of a mixture of fluorescent secondary antibodies: Alexa Fluor^®^594 Donkey anti-Mouse (Thermo Fisher Scientific A21203, 1:300), Alexa Fluor^®^633 Donkey anti-Goat (Thermo Fisher Scientific A21082, 1:300) and Streptavidin, Alexa Fluor™ 488 conjugate (Thermo Fisher Scientific S11223, 1:300) with the inclusion of Hoechst (Sigma 14,530, 5 µM). After 1 h, sections were washed with PBS and distilled water and mounted with Mowiol (Calbiochem #475,904). Imaging was performed on a Zeiss LSM 780 confocal laser scanning microscope using a 20 × Plan Apochromat NA = 0.8 air objective or a 40 × Plan-Neofluar NA 1.3 oil-immersion objective with 405, 488, 568 and 647 nm lasers and appropriate filters (Carl Zeiss B.V., Sliedrecht, the Netherlands).

### Gene sets from GWAS studies and literature

AD-associated GWAS risk genes were extracted from the NHGRI-EBI catalog on November 27, 2019 [7]. Data from four traits were downloaded: ‘Alzheimer’s disease’ (EFO-0000249), ‘Alzheimer’s disease biomarker measurement’ (EFO-0006514), ‘p-tau measurement’ (EFO-0004763) and ‘late-onset Alzheimer’s disease’ (EFO-1001870). Intergenic regions and duplicates were removed and the resulting gene set was intersected with 1112 genes of the human microglia core profile of [9] that were expressed in the present dataset. This resulted in 63 GWAS AD-risk genes that are present in the microglia core profile.

Comparison with previously published snRNAseq of human AD brain tissue was performed by extracting relevant gene sets and plotting their expression in heatmaps. From [11], ‘supplementary table 9′ was downloaded and markers of clusters ‘m1′ and ‘m2′ (AD-enriched) with a logFC > 0 were used, resulting in a geneset of 22 genes. From [22], a gene set from ‘supplementary table 2′ (‘Mic’ sheet, differentially expressed genes between AD and non-AD donors) was extracted and genes with an adjusted *p*-value < 0.01 were used, resulting in 47 genes.

Gene sets from two mouse studies were compared. (1) From Keren-Shaul et al. (2017) [18], Table S2 was downloaded. The top 100 most significant genes upregulated in ‘Microglia3′ were extracted and ribosomal genes were removed from the list, as these are barely expressed in nuclei, resulting in a gene set of 40 genes. 2) From [30], dataset EV7 was downloaded and the top 100 significantly (*p* < 0.01) upregulated genes in ARM vs HM.1 cells within APPtg mice were extracted. Ribosomal genes were removed from the list, resulting in a gene set of 37 genes.

### Quantification of amyloid-β and tau load

For quantification of amyloid-β and phospho-tau load, stained sections from the neuropathological evaluation, as described above, were obtained from the NeuroBiobank of the Institute Born-Bunge (NBB-IBB), Wilrijk (Antwerp), Belgium (ID: BB190113). Imaging was performed using a Hamamatsu Nanozoomer. Amyloid-β and phospho-tau load were quantified using ImageJ. Snapshots were made of each sample on a representative grey-matter area of 2 mm^2^ with an 8 × magnification. First, the image was split into three separate color channels. The blue channel was used for thresholding to remove background from the hematoxylin staining. Then, the fraction of positive pixels was used as a measure for amyloid-β or tau pathology.

### Image analysis ITGAX and P2RY12

Acquired TMA images were processed using FIJI (ImageJ v1.53c). After tissue detection, the median intensity value was used for background correction and an image set containing channel grey images was created for each tissue core. A microglia mask based on ITGAX and P2RY12 was generated using the Otsu’s automatic image thresholding method. For each core, total tissue area and area covered by microglia (P2RY12 and/or ITGAX) were calculated and mean intensity within the regions of interest (mask) was calculated.

### Bulk RNAseq data analysis

Data pre-processing was performed with the Lexogen Quantseq 2.3.1 FWD UMI pipeline on the BlueBee Genomics Platform (1.10.18). Bam files were used as input for htseq-count [2] and reads mapping to both intronic and exonic regions were counted. Count files were loaded into R and analyzed with edgeR and/or DESeq2 [20, 28]. Principal component analysis was performed on the VST transformed counts obtained by DESeq2. Differential gene expression analysis was performed with both edgeR and DESeq2. Visualizations were made with the CRAN package ‘ggplot2’.

### snRNAseq data analysis

Raw reads were processed using Cell Ranger 3.0.0 with default settings, the pre-mRNA package and aligned to the human GRCh38 genome. From the bam file, exonic reads and intronic reads mapping in the same direction as the mRNA were counted per barcode with Abacus to distinguish barcodes containing nuclear RNA from ambient and cytoplasmic RNA [37]. The following thresholds were used: 1) > 100 exonic reads; 2) > 250 intronic reads; 3) intronic reads > exonic reads to make sure the dataset consisted only of nuclei and no cellular debris (Figure S1c). The counts corresponding to these barcodes were extracted from the raw count matrix generated by Cell Ranger and loaded in R with Seurat (3.0.3). Nuclei with a mitochondrial content > 5% were removed from the dataset. Scrublet was used to filter out doublets [36]. Estimated doublet rate was set on 10%, in line with multiplet rates described in the 10 × Genomics user guide. Count matrices of the three brain regions per donor were merged into one file per donor using the ‘merge’ function. The data were log normalized using the ‘NormalizeData’ function in Seurat. Highly variable features (HVGs) were determined using the VST method. The datasets from the different donors were anchored and integrated with default settings using reciprocal PCA [32]. The data were scaled and heterogeneity associated with number of UMIs, gender and mitochondrial content was regressed out and the data were clustered using the graph-based clustering approach implemented in Seurat with a resolution of 0.15. Then, separate objects were made for each cell type and the analysis was rerun on each cell type individually, now using canonical correlation analysis instead of reciprocal PCA and additionally regressing out ribosomal content. Subclustering and dimensionality reduction were performed with Seurat with default settings. To determine an appropriate cluster resolution for each cell type, the number of obtained clusters per resolution in a range of 0–2 (steps of 0.1) was plotted against each other, and a resolution was chosen where there was a plateau in the plot. Additionally, enriched genes per subcluster were determined using differential gene expression analysis and if subclusters did not have any enriched genes (logFC > 0.5, *p*.adjust < 0.05), the data were clustered with a lower resolution. (Sub)clusters containing markers of multiple cell types were removed. Differential gene expression analysis between (groups of) (sub)clusters was performed with logistic regressions with donor as a latent variable on the unintegrated normalized counts. (Sub)cluster distribution was calculated per sample as (number of nuclei in each cluster)/(total number of nuclei)*100. Chi-squared statistics were performed with the ‘chisq.test’ function in R. ANOVAs were performed with the ‘anova_test’ function from the rstatix package. Comparisons between amyloid-β/tau load and (sub) cluster distribution were performed using Pearson correlations with the ‘cor.test’ function. FDRs were calculated using the ‘*p*.adjust’ function. Gene ontology analysis was performed on significantly differentially expressed genes (*p* < 0.05 and logFC > 0.15) using clusterProfiler with a *p*- and *q*-value cutoff of 0.05. Average gene expression per cluster was calculated with the ‘AverageExpression’ function from Seurat. Trajectory analysis was performed with Monocle3 [27]. First, we extracted the homeostatic- and AD1-microglia from the dataset, then we rescaled the data as described above and projected the data onto a UMAP. The dimensionality reduction, feature loadings and clustering derived from Seurat were used as input for Monocle3. Differential gene expression analysis of the HVGs over pseudotime was performed with the ‘graph_test’ function on the raw counts. Average gene expression per pseudotime-bin was visualized in a heatmap. Visualizations were made with the CRAN packages ggplot2 and gplots.

## Results

### Enrichment of less abundant CNS cell types by depletion of neurons and oligodendrocytes/OPCs

Amyloid-β and tau-associated AD-changes were studied in single-nucleus transcriptomes by FACS sorting of DAPI^pos^ nuclei from 10 AD and 8 non-demented controls, and processed on a 10× Genomics platform (Fig. 1a–c). As inclusion criteria, AD donors with only amyloid-β pathology (no tauopathy yet) in the occipital cortex (OC) and both amyloid-β and tau pathology in the occipitotemporal cortex (OTC) were selected for this study. The corresponding brain regions were analyzed in non-demented controls. Control donors were divided into two groups: CTR donors without any detectable amyloid-β or tau pathology; CTR+ donors with low levels of amyloid-β deposition in both regions, but no detectable tau (Table S1). 90% of the isolated nuclei were either derived from neurons (NEUN^pos^) or oligodendrocytes/OPCs (OLIG2^pos^) (Fig. S1a). To enrich for less abundant nuclei populations, neuronal and oligodendrocyte/OPC nuclei were depleted, yielding 482,472 NEUN^neg^OLIG2^neg^ nuclei in the snRNAseq dataset. A median of 1,052 unique genes per nucleus was detected, of which only 0.1% were mitochondrial and 0.3% ribosomal, indicating high quality of the purely nuclear population (Fig. S1b, S1c).

To identify distinct cell types, unsupervised, gene-expression-based clustering of the NEUN^neg^OLIG2^neg^ nuclei was performed, resulting in 12 distinct clusters that were present in all samples (Fig. S1d and e, Table S2). The largest cluster, about 40% of the nuclei, was enriched for microglia-specific gene expression (*P2RY12*, *CSF1R*, *CX3CR1*; *n* = 148,606). The two second largest clusters, 30% of the nuclei, consisted of astrocytes (*n* = 128,764) that were enriched for *GFAP*, *SLC1A2*, *ATP1B2* and *AQP4* expression. Because of the depletion strategy, less abundant CNS cell types were also identified (Fig. 1d, e, Fig. S1d and e, Table S2), namely endothelial cells enriched in *CLDN5* expression (*n* = 26,957), CNS-associated macrophages [16] enriched in *CD163*, *SIGLEC1*, *MRC1* and *LYVE1* expression (*n* = 17,979, Fig. 1f), lymphocytes enriched in *IL7R* and *MS4A1* expression (*n* = 12,675) and mural cells (pericytes and smooth muscle cells; [33]) enriched in *PDGFRB* and *ACTA2* expression (*n* = 12,396). Additionally, a cluster containing fibroblasts [33], enriched in *COL1A1*, *DCN* and *PDGFRA* expression (*n* = 22,238), was identified. One cluster remained unidentified. UMAPs depicting gene expression levels of marker genes of each cell type are presented in Figure S2. All cell types were detected in approximately equal ratios between the donor groups and brain regions (Chi-squared, *p* > 0.05, Fig. S1d and e).

To confirm the cellular identity of NEUN^pos^ and OLIG2^pos^ nuclei, sorted populations were analyzed by bulk RNAseq (Figure S3). As expected, these nuclei abundantly expressed known marker genes for neurons (*RBFOX3* and *MAP2*), or oligodendrocytes and OPCs (*MOBP*, *PLP1*, *OLIG1* and *PDGFRA*), respectively. Astrocyte- or microglia-specific gene expression (*ALDH1L*, *AQP4*, *GFAP*, *CD74*) was depleted (Fig. S3b and g). Microglia marker genes *P2RY12*, *CX3CR1*, *TMEM119* and *HEXB* were not detected in any of the NEUN^pos^ or OLIG2^pos^ samples, confirming that only neurons and oligodendrocytes/OPCs were targeted by the depletion strategy. Although, regional differences were observed in the NEUN^pos^ population, no consistent AD-associated or age-associated changes were identified in either NEUN^pos^ or OLIG2^pos^ nuclei by bulk RNAseq (Fig. S3c, d, e, h, i and j).

In the NEUN^neg^OLIG2^neg^ snRNAseq dataset, each of the seven identified cell type clusters was analyzed individually, resulting in 7–14 subclusters (Table S3). To evaluate if donor groups differentially contributed to these subclusters, associations between donor groups and relative subcluster distribution were determined within each cell type using Chi-squared tests. For the majority of cell types, we did not find regional- or AD-associated changes in subcluster distribution or gene expression (Endothelial cells, Fibroblasts, Mural cells, CNS-associated macrophages and Lymphocytes; Fig. 1g, Fig. S4). In the astrocyte population, clear regional differences in subcluster distribution, but no AD-associated changes, were identified (Supplementary text, Fig. 1g, Fig S5, Fig S6a, Table S4). Only in the microglia population, subcluster distribution was significantly associated with donor groups (CTR vs AD) in the OC (*p* = 0.01, for OTC *p* = 0.07) (Fig. 1f, Fig. S6a). These results indicate that, in this dataset, only the expression profile of microglia is significantly affected by AD pathology. Next, we focused on AD-associated changes in microglia.

### Two groups of microglia subclusters are associated with AD

**Fig. 2:**
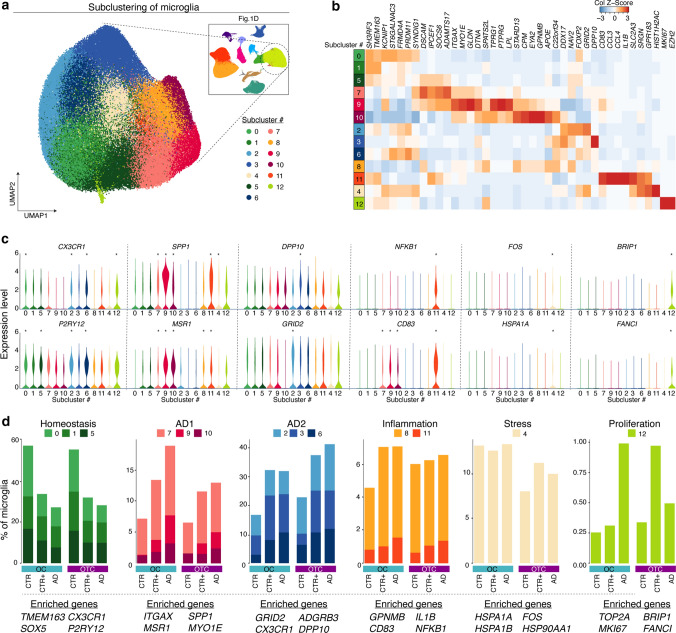
Two groups of microglia subclusters are associated with AD. **a** UMAP of 148,606 microglia nuclei in 13 subclusters. **b** Heatmap depicting average expression of three most enriched genes per subcluster. **c** Violin plots depicting expression of selected genes per subcluster. *: significantly enriched genes for each subcluster compared to all other subclusters (logFC > 0.15, adjusted *p*-value < 0.05). **d** Bar plots depicting the percentage of microglia in each subcluster group by category. Representative marker genes are listed on the bottom. *OC* Occipital Cortex; *OTC* Occipitotemporal Cortex; *CTR* non-demented controls; *CTR+ * non-demented controls with mild amyloid-β pathology; *AD* clinical and neuropathological Alzheimer’s disease

Within the microglia population, 13 distinct subclusters were identified (Fig. 2a, b). Variation between samples within donor groups and between brain regions was very limited, and no donor- or brain-region-specific subclusters were identified (Fig. S6c). To annotate microglia subclusters, differential gene expression analysis (DE) was performed for each subcluster compared to all others (Fig. 2c and Table S3). Subclusters were grouped into categories based on three criteria: 1) relative enrichment/depletion in AD samples; 2) similar marker gene expression; 3) branching in the trajectory analysis and location in the UMAP (Fig. 2d, Fig S6b, c, d). Subclusters 0, 1 and 5 were more abundant in CTR than in AD samples and contained homeostatic microglia, as these cells were enriched for homeostasis markers, such as *P2RY12* and *CX3CR1*. Subclusters 7, 9 and 10 were enriched for expression of genes detected in phagocytic/activated microglia derived from amyloid mouse models, including *ITGAX*, *LPL*, *GPNMB*, *MYO1E* and *SPP1* [18, 19]. The fraction of total nuclei in these subclusters was highest from AD, intermediate in CTR+ samples, and lowest in samples from CTR donors (Fig. 2d). Together, the microglia in these three subclusters were termed “AD1” microglia. Subclusters 2, 3 and 6 were enriched for expression of homeostasis genes, such as *CX3CR1* and *P2RY12*, but also enriched for several neuron-related genes, such as *GRID2*, *ADGRB3* and *DPP10*. These subclusters were also more abundant in CTR+ and AD samples than in CTR samples, and denoted as “AD2” microglia. Two smaller subclusters (8 and 11) were associated with (pro-)inflammatory responses. Subcluster 8 was enriched in microglia-specific gene expression but also genes often associated with other macrophages (e.g. *CD163*), and subcluster 11 was enriched for gene expression related to the NF-κB pathway (*IL1B* and *NFKB1*). Subcluster 4 was enriched for expression of several early-response genes (*FOS*, *JUNB*) and heat-shock genes (*HSPA1A* and *HSPA1B*), indicative of cellular stress. A small subcluster of microglia was enriched for expression of genes associated with proliferation, such as *TOP2A* and *MKI67* (cluster 12), and these were also more abundant in AD samples than in CTRs (Fig. 2b–d, Fig S6c and d, and Table S3).

Taken together, heterogeneity of microglia in the human brain was identified and AD was associated with substantial transcriptional changes. In CTR+ and AD samples, abundance of homeostatic subclusters was consistently reduced and occurrences of AD1- and AD2-profiles were increased.

### AD1-microglia subclusters gradually transition towards a phagocytic/activated profile

**Fig. 3:**
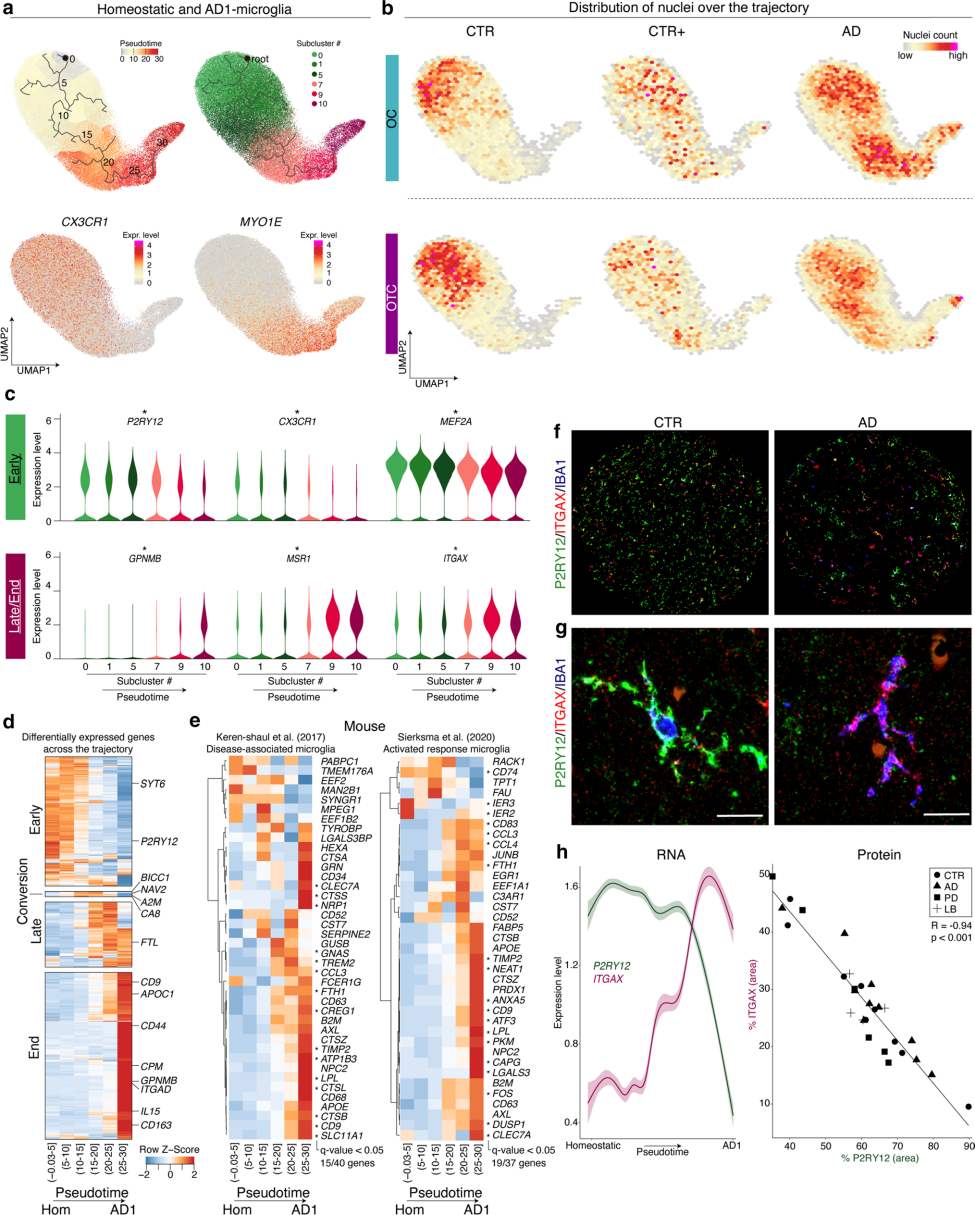
AD1-microglia subclusters gradually transition towards a phagocytic/activated profile**.** **a** UMAPs depicting trajectory analysis of homeostatic and AD1-subclusters. Color-scale indicates pseudotime, subclusters and expression of *CX3CR1* (homeostasis) and *MYO1E* (AD1). **b** Density heatmaps depicting the distribution of nuclei over the UMAP for each sample group. **c** Violin plots depicting expression of selected genes per subcluster. *: genes significantly differentially expressed (Moran’s I test, *q*-value < 0.05). **d** Heatmap depicting all genes significantly differentially expressed over the trajectory *: Moran’s I test, *q*-value < 0.05). **e** Heatmaps depicting top 40 (non-ribosomal) DAM genes from [18] and top 37 (non-ribosomal) ARM genes from [30] over pseudotime. *: Moran’s I test, *q*-value < 0.05. **f** IBA1, P2RY12, and ITGAX co-expression in tissues from a CTR and an AD donor. Tissue core diameter = 1 mm. **g** Left: Microglia expressing P2RY12 and IBA1, but not ITGAX. Right: Microglia expressing IBA1 and ITGAX, but not P2RY12. Microglia are from the tissue section and exist next to each other. Scale bar = 20 μm. **h** Left: Gene expression of P2RY12 (green) and ITGAX (pink) along the trajectory. Right: Correlation between % P2RY12^pos^ area and % ITGAX^pos^ area per sample. *OC* Occipital Cortex; *OTC* Occipitotemporal Cortex; *CTR* non-demented controls; *CTR+ * non-demented controls with mild amyloid-β pathology; *AD* clinical and neuropathological Alzheimer’s disease; *PD* Parkinson’s disease; *LB* Lewy body dementia

To determine the relationship between the homeostatic and AD1/2-microglia subclusters, a trajectory analysis was performed (Fig. 3a). Microglia were computationally ordered along a gene-expression-driven pseudotime trajectory representing a biological process associated with the transition from the homeostatic state, as seen in CTR, to the activated states apparent in CTR+ and AD. For the transition from homeostatic to AD2-microglia (clusters 0, 1, 5, 2, 3, 6), the trajectory was very short and the AD2 subcluster did not appear as intermediate stages of each other (data not shown). In contrast, for AD1-microglia, pseudotime analysis ordered the homeostasis and AD1-subclusters on the trajectory as follows: 0, 1, 5, 7, 9, 10 (Fig. 3a). While microglia from the CTR donors were mainly located at the start of the trajectory, microglia from the AD donors were mostly present at the end (Fig. 3b). Differentially expressed genes over the trajectory were determined using spatial autocorrelation analysis (Fig. 1c, d, Table S5). Four groups of genes were identified: “Early” genes that were abundantly expressed at the beginning of the trajectory and decreased over time; “Conversion” genes that were exclusively expressed in the middle of the trajectory; “Late” genes that were abundantly expressed later in the trajectory but depleted near the end; and “End” genes that were exclusively abundant at the end of the trajectory (Fig. 3d, Table S6).

To compare the AD1-microglia trajectory with the phagocytic/activated microglia profile reported in amyloid mouse models, expression of enriched DAM (disease-associated microglia, [18]) and ARM (activated-response microglia, [30]) genes was visualized over the trajectory. Of both gene sets, the expression of nearly all genes increased over time in the AD1-trajectory, indicating that AD1 microglia are similar to the phagocytic/activated profile previously identified in amyloid mouse models (Fig. 3e).

To confirm the AD1-microglia trajectory in situ, immunohistochemistry for P2RY12, IBA1 and ITGAX was performed on a human brain dementia cohort containing frontal cortex tissue from 29 age- and post-mortem-delay matched donors (Fig. 3f, g). P2RY12 was detected in all analyzed cells in a range from 40 to 90% of the microglia mask, indicating that these cells were indeed microglia. A significant inverse correlation between P2RY12 and ITGAX immunoreactivity in microglia was identified, which illustrates a consistent functional transformation of microglia in line with gene expression changes along the AD1-trajectory (Fig. 3h).

### Microglia show distinct amyloid-β and tau-associated profiles

**Fig. 4:**
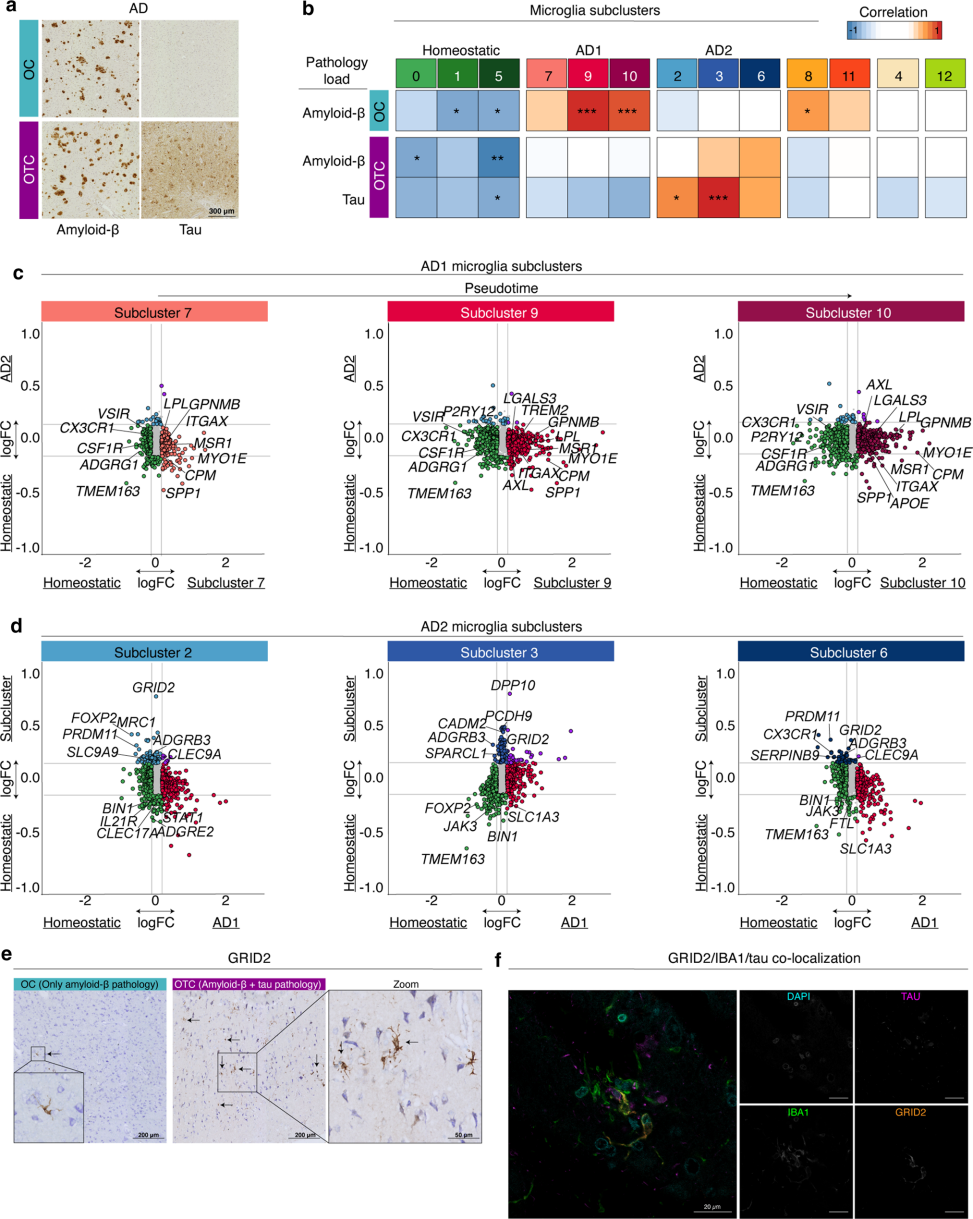
Microglia segregate into distinct amyloid-β and tau-associated profiles. **a** Amyloid-β and phospho-tau immunohistochemistry of an AD donor. **b** Heatmap depicting Pearson correlations of amyloid-β/tau load versus the percentage of microglia located in each subcluster. *: *p* ≤ 0,05; **: *p* ≤ 0.01; ***: *p* ≤ 0.001. **c** Four-way plots depicting differential gene expression of the indicated AD1 clusters (on *x*-axis) versus homeostasis (subclusters 0, 1, 5) and logFC of AD2 (subclusters 2, 3, 6) versus homeostasis (subclusters 0, 1, 5) on the y-axis. **d** Four-way plots depicting differential gene expression of AD1 (subclusters 7, 9, 10) on the x-axis versus homeostasis (subclusters 0, 1, 5) and logFC of the indicated AD2 clusters versus homeostasis (subclusters 0, 1, 5) on the y-axis. **e** GRID2 expression (brown) in AD samples with only amyloid-β or both amyloid-β and tau pathology. Cresyl violet was used to detect nuclei. **f** IBA1 (green), GRID2 (orange) and phospho-tau (magenta) colocalization in human AD brain tissue. *OC* Occipital Cortex; *OTC* Occipitotemporal Cortex

From AD donors, samples with only amyloid-β (OC) or both amyloid-β and tau pathology (OTC) were analyzed (Fig. 4a). To determine whether different microglia subtypes were associated with the degree of pathology, the level of amyloid-β and tau was quantified and correlated to the percentage of microglia in each subcluster (Fig. 4b, Fig. S6d, Fig. S7a). Strong positive correlations were observed between amyloid-β load and AD1-microglia abundance in samples that contained only amyloid-β pathology (Fig. 4b) but not in the samples that contained both amyloid-β and tau pathology (Fig. 4b, S7c). This indicates that AD1-microglia are associated with amyloid-β, but when tau pathology is present this correlation is absent. This suggests that additional presence of tau induces an additional microglia subtype. In samples that contained both amyloid-β and tau pathology (AD-OTC), significant positive correlations were detected between tau-load and AD2-microglia abundance (Fig. 4b, Fig. S7c). Additionally, negative correlations were identified between homeostasis clusters and amyloid-β and/or tau-load in both regions (Fig. 4b, Fig. S7c), suggesting a decrease in homeostatic microglia abundance in the presence of pathology. AD1 microglia abundance did not correlate with amyloid-β load in the OTC samples. This may be due to the increased abundance of AD2 microglia associated with tau pathology in these samples. As we used relative subcluster abundance as a variable, AD1 and AD2 microglia abundance are two dependent variables, and if AD2 microglia abundance increases in the OTC samples, AD1 abundance will relatively decrease and no longer (positively) correlate with amyloid-β load.

To compare AD1 and AD2 microglia profiles, differential gene expression analysis of AD1/AD2 versus homeostasis subclusters was performed (Fig. 4c, d, Table S7). All three AD1-subclusters were significantly different from homeostatic microglia, and ~ 2000 differentially expressed genes were identified (Fig. 4c; 218, 652, 827 genes enriched and 218, 724 and 620 genes depleted in subclusters 7, 9 and 10, respectively). Of the AD1-subclusters, subclusters 9 and 10 were more different from homeostatic microglia than subcluster 7, as seen by the higher number of identified DE genes and higher logFC values (Fig. 4c). This aligns with the findings of the pseudotime analysis, and subcluster 10 was present at the end of the trajectory and nuclei of cluster 7 were found in the middle (Fig. 3a). Gene ontology analysis indicates that AD1-microglia were associated with ‘cell migration’ (*ITGAX*, *GPNMB* and *FLT1*), ‘phagocytosis’ (*COLEC12*, *MSR1*, *AXL*) and ‘lipid localization’ (*SPP1*, *LPL*, *PPARG*). These functions are similar to the phagocytic/activated microglia profile observed in amyloid mouse models. Moreover, the GO term ‘cellular response to amyloid-beta’ was significantly enriched in subclusters 9 and 10, in line with our finding that AD1 microglia abundance is associated with amyloid-β load (*CACNA1A*, *NAMPT*, *TREM2*) (Fig. S7b, Table S8).

AD2 microglia were less different from homeostatic microglia and ~ 150 significantly DE genes were identified (Fig. 4d; 80, 89, 45 genes enriched in subclusters 2, 3 and 6, respectively; 99, 139, 79 genes depleted, respectively; Table S7). Gene ontology analysis indicated that AD2 microglia possibly have neurotrophic functions, such as ‘synapse organization’ (*GRID2*, *ADGRB3*, *GPM6A*) and ‘axonogenesis’ (*UNC5C*, *SLIT2*, *NRXN1*) (Fig. S7d, Table S8). The most enriched gene in AD2 microglia was GRID2. GRID2 expressing cells had a microglia-like morphology, and were abundantly present in AD samples with tau pathology but rarely identified in CTR and AD samples without tau pathology (Fig. 4e, Fig S8a and b). Immunofluorescent triple labelling for GRID2, IBA1 and tau on enthorinal allocortex and temporal isocortex of 8 CTR and 12 AD donors showed that GRID2 cells were abundant in AD samples with increasing amounts of tau pathology and localized to regions with tau pathology (Fig. 4f, S8c–f). Also, GRID2^pos^ microglia were often observed in (neuritic) plaque-like structures, indicating that this microglial subtype was associated with tau pathology (Figure S8b, g and h).

In summary, these results indicate that AD1 microglia are associated with a direct response to amyloid-β circulating in the extracellular space and that AD2 microglia are a response to phospho-tau bearing (dying) neurons. AD1 and AD2 microglia gene expression profiles did not overlap, and only a few genes were shared by both profiles, supporting that these microglia have distinct phenotypes.

## Discussion

Here, snRNAseq of 482,472 NEUN^neg^OLIG2^neg^ nuclei from control and AD brain tissue is reported, to determine the effect of pathological changes associated with amyloid-β and tau pathology on gene expression in microglia. To capture the changes associated with disease progression, we included two brain regions per donor, one region with only amyloid-β pathology, and one containing both amyloid-β and tau pathology. Of seven included cell types, AD-associated changes in gene expression were only detected in microglia. Due to the used depletion strategy, high numbers of microglia and astrocyte nuclei were obtained, but lower numbers of the other cell types. This might have precluded the detection of possible subtle AD-associated gene expression changes in depleted cell types. In our data, in astrocytes only regional differences in subcluster distribution were detected. It is possible that AD-associated changes in astrocytes are more pronounced in white matter, and were not detected as we exclusively analyzed grey-matter tissue. This may also be the case for the other cell types we analyzed.

Recently, two snRNAseq studies of unsorted human AD brain nuclei were reported that suggested AD-associated changes in microglia. In both studies, a set of differentially expressed genes between AD donors and controls was identified. Mathys et al. (2019) [22] and Grubman et al. (2019) [11] identified 47 and 22 AD-associated genes, respectively (Fig. S7e and f). Using our enrichment strategy, we increased the number of analyzed microglia to an average of 9000 per donor. This allowed us to capture a much larger spectrum of microglia heterogeneity and increase statistical power, resulting in the identification of ~ 2500 AD-associated differentially expressed genes in microglia.

Two distinct AD-associated microglia profiles were identified that associated with either amyloid-β (AD1) or hyperphospho-tau (AD2). AD1-microglia share features with phagocytic/activated microglia that associated with amyloid-β plaques in amyloid mouse models [8, 18, 30]. In mice, the transition to a phagocytic/activated microglia phenotype is Trem2 dependent [18]. In our AD1-microglia trajectory, *TREM2* expression was detected prior to *APOE* (Figs. 3e and 4c), suggesting a similar response to amyloid-β. Recently, Nguyen et al. (2020) showed that CD163 is nearly exclusively present in amyloid-associated microglia [25]. Indeed, in our AD1-microglia trajectory, *CD163* is significantly differentially expressed and exclusively enriched in nuclei at the end of the AD1-trajectory (Fig. 3d, Table S3, S5, S6).

In sporadic AD, genome-wide association studies (GWAS) identified several risk loci and genes located on these loci are expressed in immune-related tissues and cell types [14]. Of the 63 AD-risk genes [7] expressed in human microglia [9], 15 were significantly enriched and highly expressed in AD1-microglia, and six genes were moderately enriched in AD2-microglia (Fig. S7g). This finding is in line with a recent mouse study of Sierksma et al. (2020), where it was shown that the genetic risk of AD is functionally associated with the microglia response to amyloid-β pathology and not to phospho-tau pathology, suggesting that amyloid-β pathology is upstream of tau pathology [30]. This indicates that the immune response of AD1-microglia to amyloid-β pathology is involved in the onset and progression of AD.

The abundance of AD1-microglia significantly correlated with amyloid-β load, but this effect was only present in samples without detectable tau pathology. AD2-microglia were more abundant in CTR+ and AD samples and associated with phospho-tau in the AD samples. Pseudotime analysis and differential gene expression analysis indicated that they represent separate populations that both originate from homeostatic microglia. The conversion of homeostatic microglia into AD1 microglia was most prevalent in the OC samples (amyloid-β). In the OTC samples (amyloid-β + tau), less homeostatic microglia converted into AD1 and a larger proportion of AD2-microglia was present. Our data suggest that the AD1- and AD2-microglia represent separate populations and we observed no evidence for reciprocal conversion.

*GRID2* expression by AD2-microglia was confirmed using immunohistochemical staining. GRID2 is a glutamate receptor, in the cerebellum also expressed by Purkinje neurons [3]. Our dataset did not contain Purkinje neuron nuclei as it was generated with cerebral brain tissue exclusively. Moreover, the GRID2^pos^ cells in the OC and OTC brain region had a microglia-like morphology. These data indicate that in these cortical brain areas, AD2 microglia expressed GRID2 (Fig. 4e, S8). AD2-microglia were already detected prior to overt tau pathology, and still were quite similar to homeostatic microglia. In mice, microglia (pathologically) prune synapses in response to phospho-tau, which is regulated by the complement system [13]. However, complement-associated gene expression was not enriched in AD2-microglia. Their partially homeostatic signature and lack of complement activation might reflect a potential neurotrophic function of AD2-microglia in response to neuronal stress. The AD2-microglia phenotype could be of great interest for future studies, as the degree of tau pathology correlates with the degree of dementia in AD donors, whereas the degree of amyloid-β does not [24].

Taken together, here we report for the first-time distinct profiles of microglia associated with either amyloid-β (AD1) or tau pathology (AD2). AD1-microglia are similar to the phagocytic/activated profile identified in amyloid mouse models. AD2-microglia have not been identified before and might be tissue supportive or responsive to neuronal loss. These multiple microglia phenotypes in human AD CNS may offer new targets for microglia-state-specific therapeutic strategies.

## Supplementary Information

Below is the link to the electronic supplementary material.

Fig. S1. FACS strategy and quality control. **a** Nuclei were selected as DAPIpos events. Autofluorescent events were gated out with unused channels. The nuclei population was depleted for neurons and oligodendrocytes/OPCs by negative gating of NEUNpos and OLIG2pos nuclei. snRNAseq was performed on the NEUNnegOLIG2neg population. **b** Violin plots depicting number of expressed genes per nucleus and the percentages of expressed genes that are mitochondrial and ribosomal. **c** Barcode filtering strategy. X-axis depicts the number of exonic UMI counts per barcode, Y-axis the number of intronic UMI counts per barcode. Barcodes with exonic > intronic are assumed to be (partial) cells. In the analysis, barcodes that have > 250 intronic counts, > 100 exonic counts and intronic > exonic were included. **d** Bar plots depicting relative abundance per cell type as a fraction of the total NEUNnegOLIG2neg population per group. Circles represent individual samples. Bar indicates mean with standard error. **e** Distribution of cell types in the NEUNnegOLIG2neg population of each sample in bars. OC = Occipital Cortex; OTC = Occipitotemporal Cortex; CTR: non-demented controls; CTR+: non-demented controls with mild amyloid-β pathology; AD: clinical and neuropathological Alzheimer’s disease. Fig S2. Cell type marker gene expression. UMAPs depicting gene expression levels of manually selected marker genes for each of the cell types depicted in Figure 1c. Fig. S3. Bulk RNAseq of OLIG2pos and NEUNpos nuclei confirms neuronal and oligodendrocyte lineage cell type identity. **a** Example of a FACS plot of the nuclei sorting strategy. **b** Boxplots depicting transformed expression levels of the OLIG2pos samples for cell type marker genes for neurons (MAP2, RBFOX3), astrocytes (ALDH1L, AQP4, GFAP, GJA1, MFGE8), oligodendrocytes and OPCs (MOBP, PLP1, OLIG1, OLIG2, PDGFRA), microglia (CD74), pericytes (PDGFRB) and endothelial cells (CLDN5). **c** Principal component analysis (PCA) depicting OLIG2pos samples. Colors indicate experimental groups. Shape indicates gender. **d** PCA plot depicting OLIG2pos samples. Colors indicate individual donors. **e** PCA plot depicting OLIG2pos samples. Colors indicate age of the donor. **f** Example of a FACS plot of the nuclei sorting strategy. **g** Boxplots depicting transformed expression levels of the NEUNpos samples for the same genes as mentioned in (**b**). **i** PCA plot depicting NEUNpos samples. Colors indicate experimental groups. (I) PCA plot depicting NEUNpos samples. Colors indicate individual donors. **j** PCA plot depicting NEUNpos samples. Colors indicate age of the donor. OC = Occipital Cortex; OTC = Occipitotemporal cortex; CTR: non-demented controls; CTR+: non-demented controls with mild amyloid-β pathology; AD: clinical and neuropathological Alzheimer’s disease. Fig. S4. Subcluster distribution of non-microglia cell types. **a** UMAP of 26,957 endothelial cells. Colors indicate endothelial cell subclusters. **b** Endothelial subcluster distribution per sample. **c** UMAP of 22,238 fibroblasts. Colors indicate fibroblast subclusters. **d** Fibroblast subcluster distribution per sample. **e** UMAP of 17,979 CNS-associated macrophages. Colors indicate macrophage subclusters. **f** Subcluster distribution of CNS-associated macrophages. **g** UMAP of mural cells (12,396 pericytes and smooth muscle cells). Colors indicate mural cell subclusters. **h** Subcluster distribution per sample of mural cells. **i** UMAP of 12,675 lymphocytes. Colors indicate lymphocyte subclusters. **j** Subcluster distribution of lymphocytes. OC = Occipital Cortex; OTC = Occipitotemporal Cortex; CTR: non-demented controls; CTR+: non-demented controls with mild amyloid-β pathology; AD: clinical and neuropathological Alzheimer’s disease. Fig. S5. snRNAseq of astrocytes reveals region-associated changes, but no AD-associated changes. **a** UMAP depicting 128,764 astrocyte nuclei. Colors indicate gene-expression based clustering. **b** Bar plots depicting sub cluster distribution per sample. **c** Heatmap depicting average expression per cluster of the highly variable genes. **d** UMAPs depicting expression of selected cluster enriched genes. **e** Boxplots depicting percentage of astrocytes in clusters 0, 4, 6, 10,12, 2 and 5 where significant differences between brain regions were identified by repeated measures ANOVA. **f** Four-way plot depicting log fold changes of differentially expressed genes between clusters 5 and 2 (OC-enriched) versus clusters 0, 4, 6, 10, 12 (OTC-enriched) in CTR and CTR+ samples (x-axis) versus AD samples (y-axis). Pearson correlation is 0.84, *p*-value < 0.01. **g** Violin plots depicting module scores of DEGs derived from Habib et al. (2020) per astrocyte subcluster. OC = Occipital Cortex; OTC = Occipitotemporal Cortex; CTR: non-demented controls; CTR+: non-demented controls with mild amyloid-β pathology; AD: clinical and neuropathological Alzheimer’s disease. Fig S6. Subcluster identification of microglia. **a** Heatmap depicting *χ*2 and *p*-values of associations between experimental groups and subcluster distribution within each cell type. **b** UMAP depicting trajectory analysis on microglia nuclei. **c** Bar plots depicting microglia subcluster distribution per sample. **d** Bar plots depicting relative abundance per microglia subcluster as a fraction of the total microglia population per group. Circles represent individual samples. Bar indicates mean with standard error. OC = Occipital Cortex; OTC = Occipitotemporal Cortex; CTR: non-demented controls; CTR+: non-demented controls with mild amyloid-β pathology; AD: clinical and neuropathological Alzheimer’s disease. Fig. S7. Correlations of pathology load versus percentage of microglia per cluster and comparisons to literature. **a** Methodology for the scoring of amyloid-β and phospho-tau load per sample. **b** Dot plot depicting representative significant gene ontology terms associated with differentially expressed genes between clusters 7, 9 and 10 versus homeostasis clusters (0, 1, 5). **c** Heatmap depicting correlations, *p*-values and FDR values of amyloid-β or phospho-tau load versus the percentage of microglia per cluster. **d** Bar plot depicting top 10 gene ontology terms associated with differentially expressed genes between all AD2-microglia (clusters 2, 3 and 6) versus all homeostatic microglia (clusters 0, 1, 5). **e** Heatmap depicting average normalized gene expression per cluster of a gene set derived from AD-enriched microglia clusters vs CTR-enriched microglia clusters of Grubman et al. (2019) **f** Heatmap depicting average normalized gene expression per cluster of a gene set derived from differentially expressed genes of microglia from AD donors versus non-AD donors from Mathys et al. (2019). **g** Heatmap depicting average normalized gene expression per cluster of 63 AD-related GWAS risk genes intersected with the human microglia core profile in Galatro et al. (2017). *: significantly enriched genes in a cluster compared to all other clusters (LR: p < 0.05, logFC > 0.15). OC = Occipital Cortex; OTC = Occipitotemporal Cortex. Fig. S8: Immunohistochemistry/fluorescence of GRID2pos microglia. **a** Representative DAB images of GRID2 staining in the OTC of two CTR donors. **b** Representative DAB images of GRID2 staining in the OTC of two AD donors. **c**–**f** Immunofluorescent triple labelling of TAU (magenta), IBA1 (green) and GRID2 (orange) of 6 different AD donors with different tau loads. Arrows point to GRID2pos microglia. **c**–**d** Immunofluorescent triple labelling of tau (magenta), IBA1 (green) and GRID2 (orange) in two AD donors. Arrows point to GRID2pos microglia (PDF 99757 KB)

Supplementary file2 Table S1: Donor and sample information. Table S2: Enriched genes per cell type cluster. Table S3: Enriched genes per subcluster within each cell type. Table S4: Differential gene expression analysis between OC-enriched clusters versus OTC-enriched clusters in astrocytes. Table S5: Spatial autocorrelation analysis of AD1-trajectory. Table S6: Genes belonging to the sections of the AD1 trajectory. Table S7: Differential gene expression between AD1/AD2 and homeostasis clusters. Table S8: Gene ontology analysis on enriched genes in AD1/AD2 microglia versus homeostasis clusters. (XLSX 18920 KB)

Supplementary file3 (DOCX 13 KB)

## Data Availability

The data reported in this study are available through Gene Expression Omnibus at https://www.ncbi.nlm.nih.gov/geo with accession number GSE148822.
