# Supplementary figures and images for: Distinct amyloid-β and tau-associated microglia profiles in Alzheimer’s disease
Source: Acta Neuropathol. 2021 Feb 20;141(5):681–96. doi: 10.1007/s00401-021-02263-w (PMC8043951; doi:10.1007/s00401-021-02263-w)

Figure S1

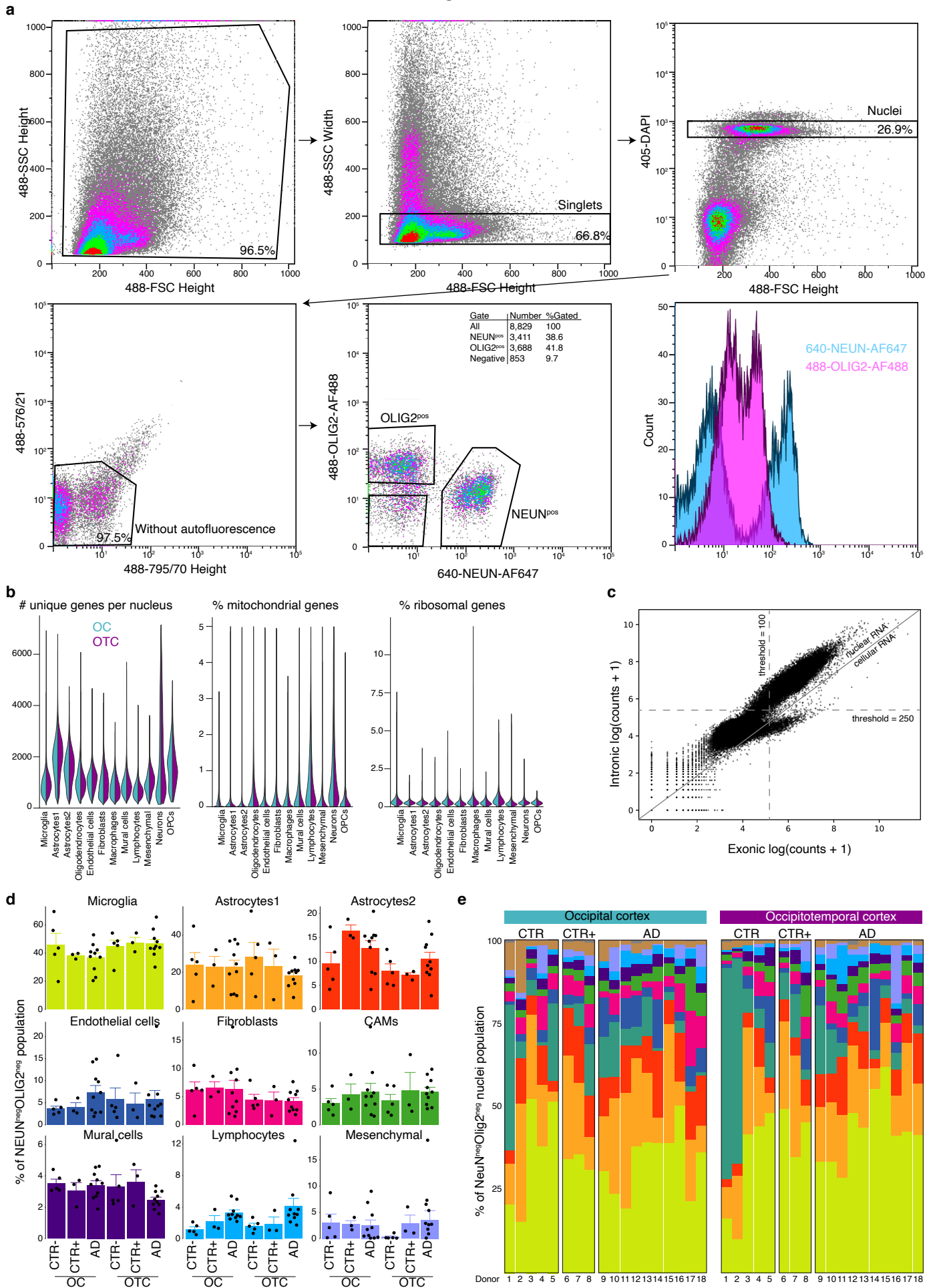

Figure S2

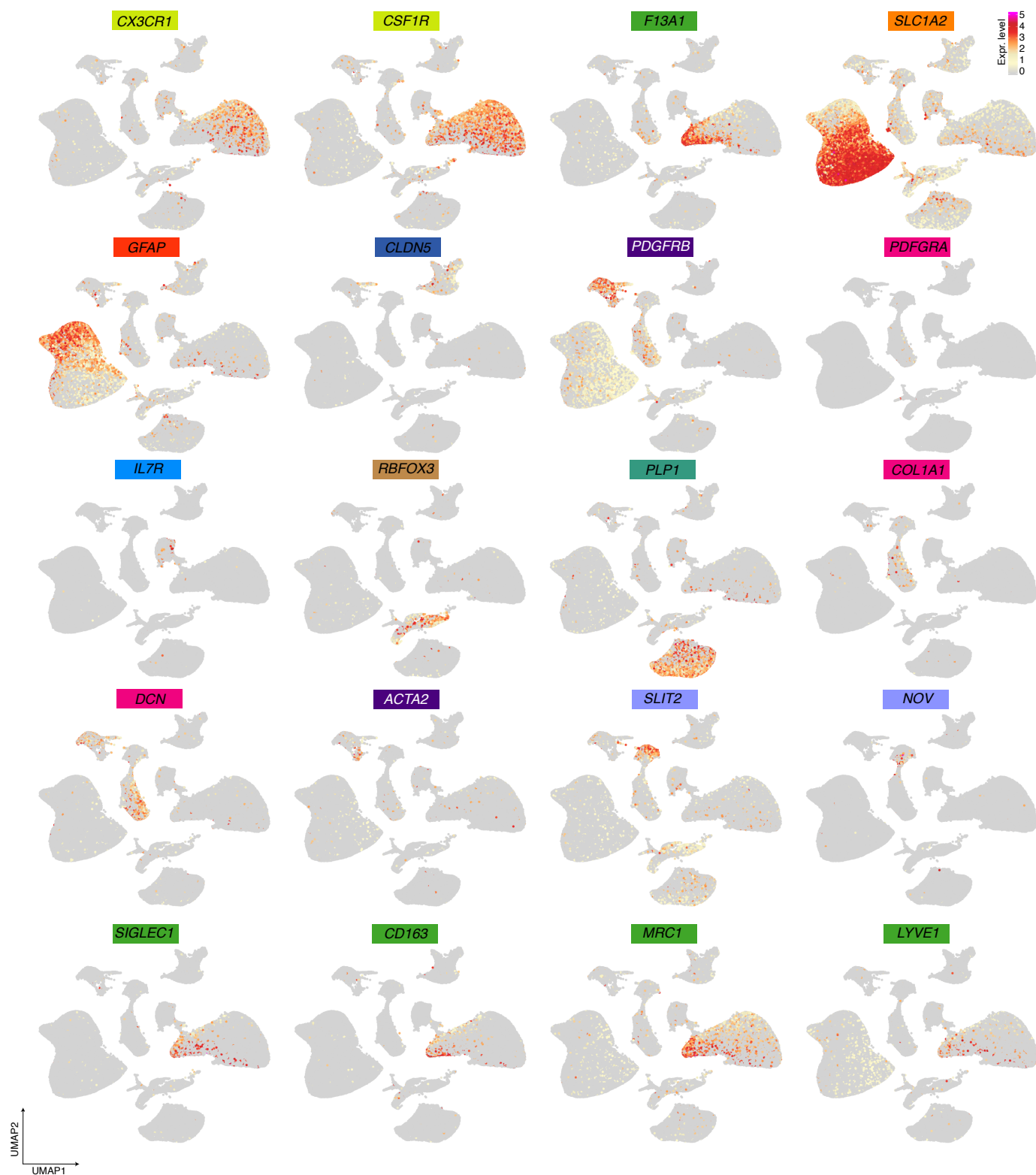

Figure S3

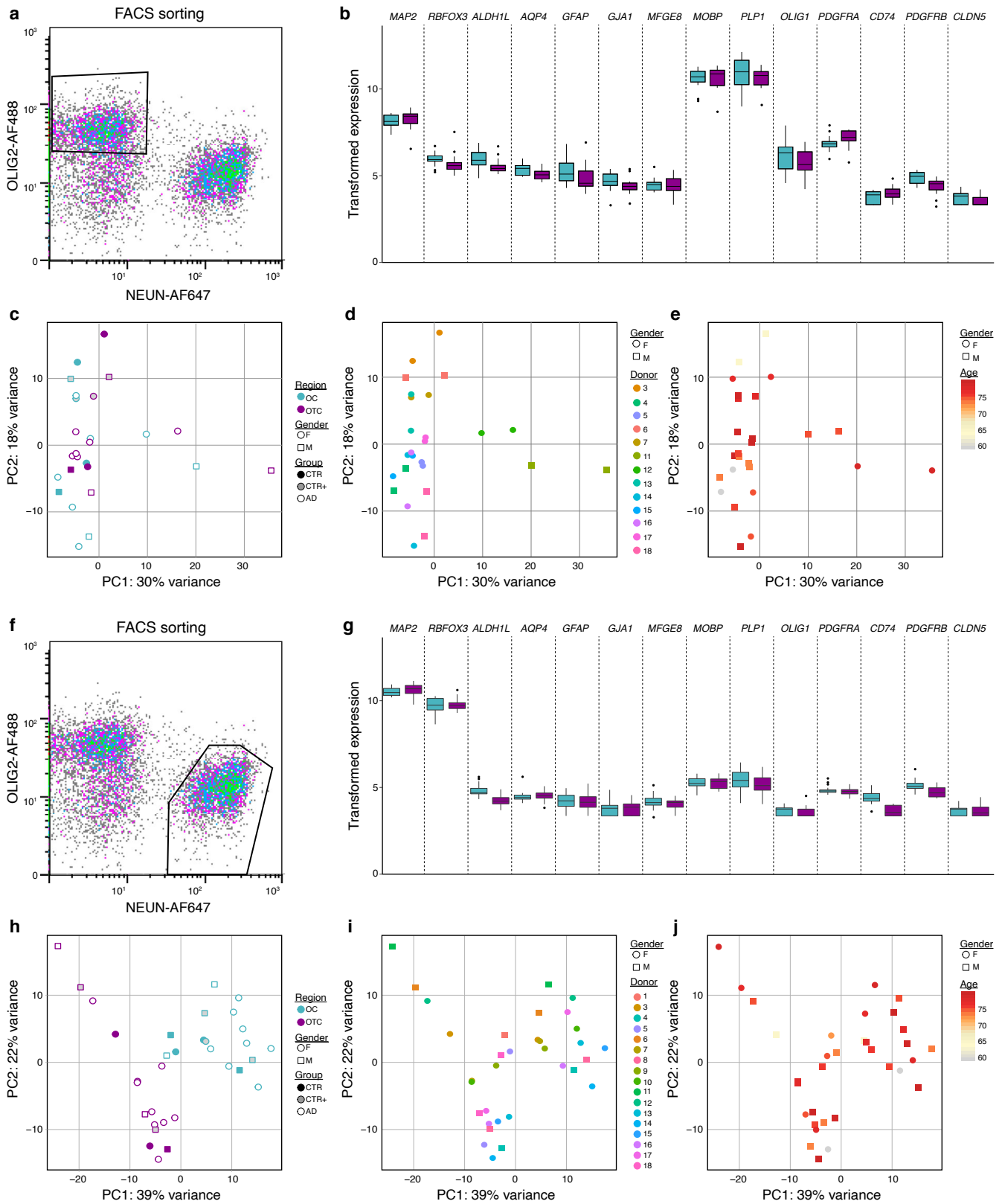

Figure S4

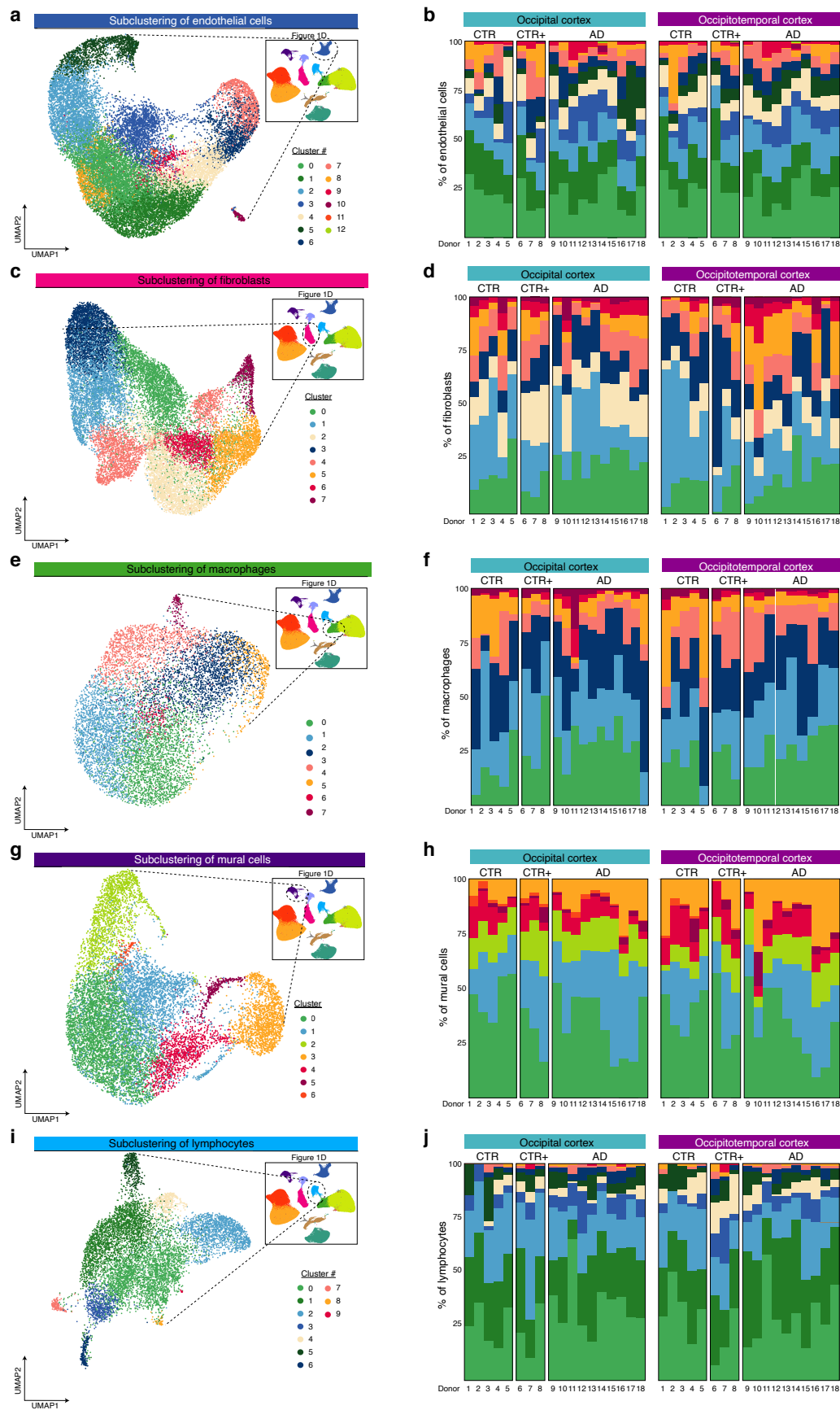

Figure S5

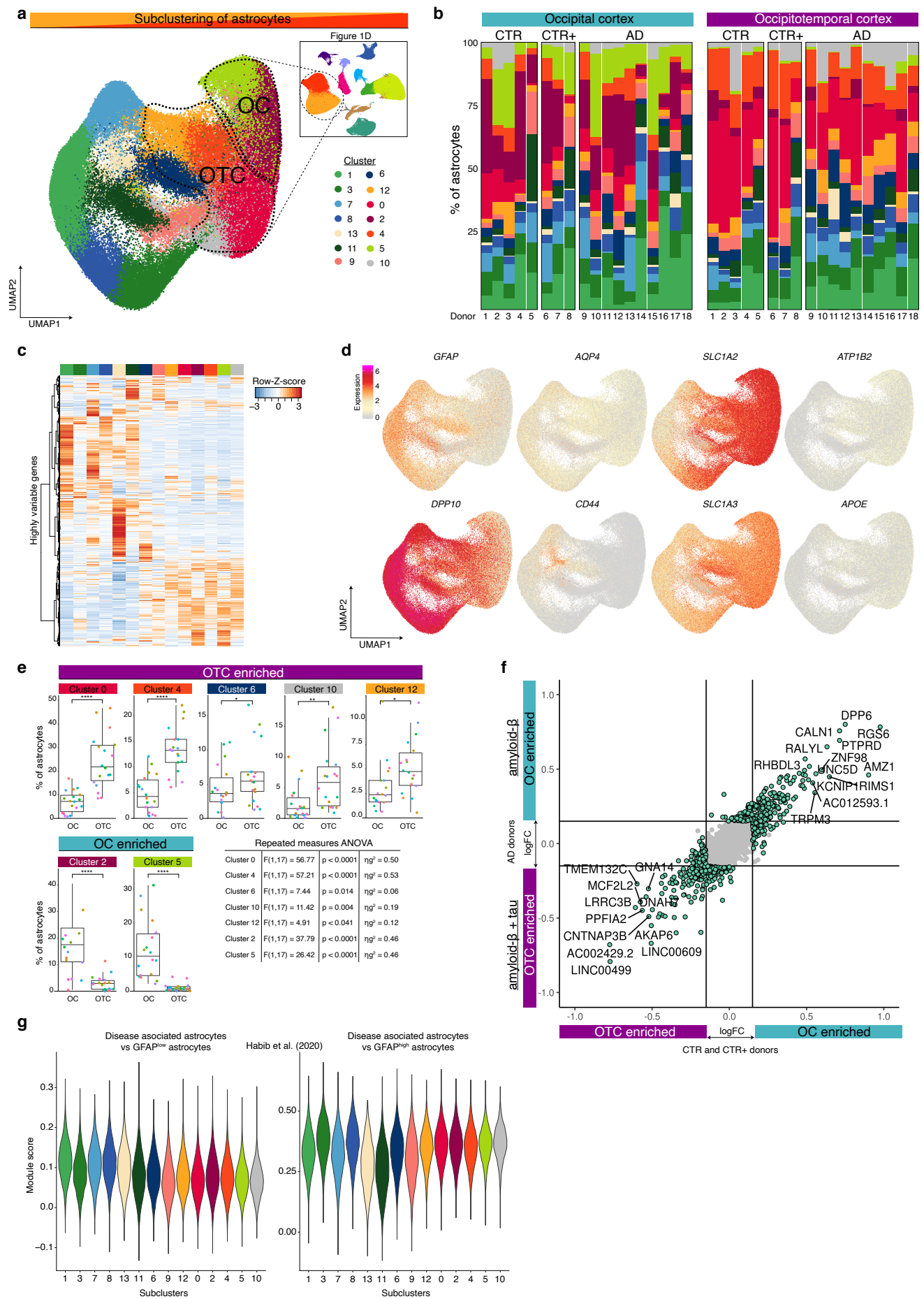

Figure S6

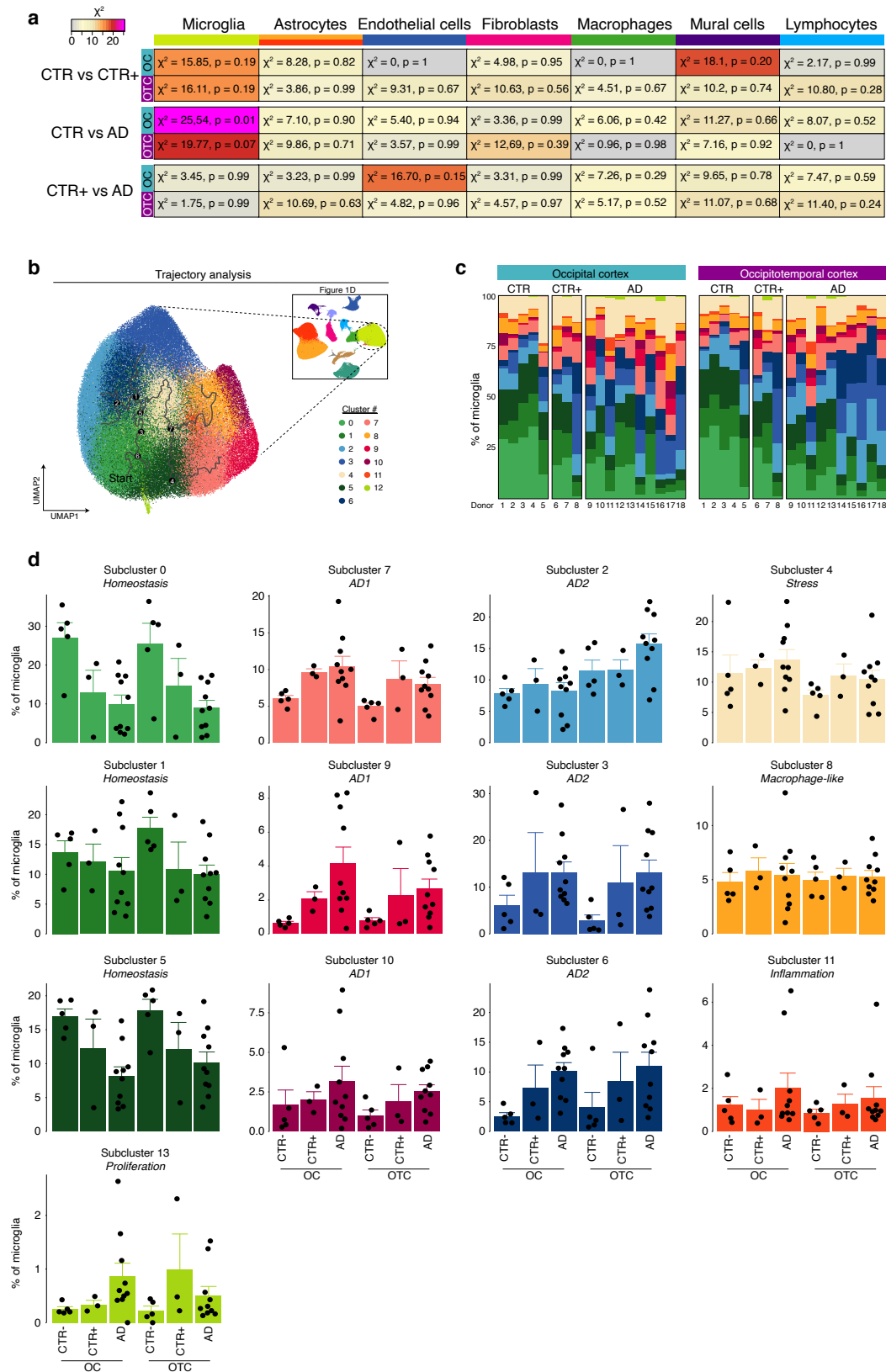

Figure S7

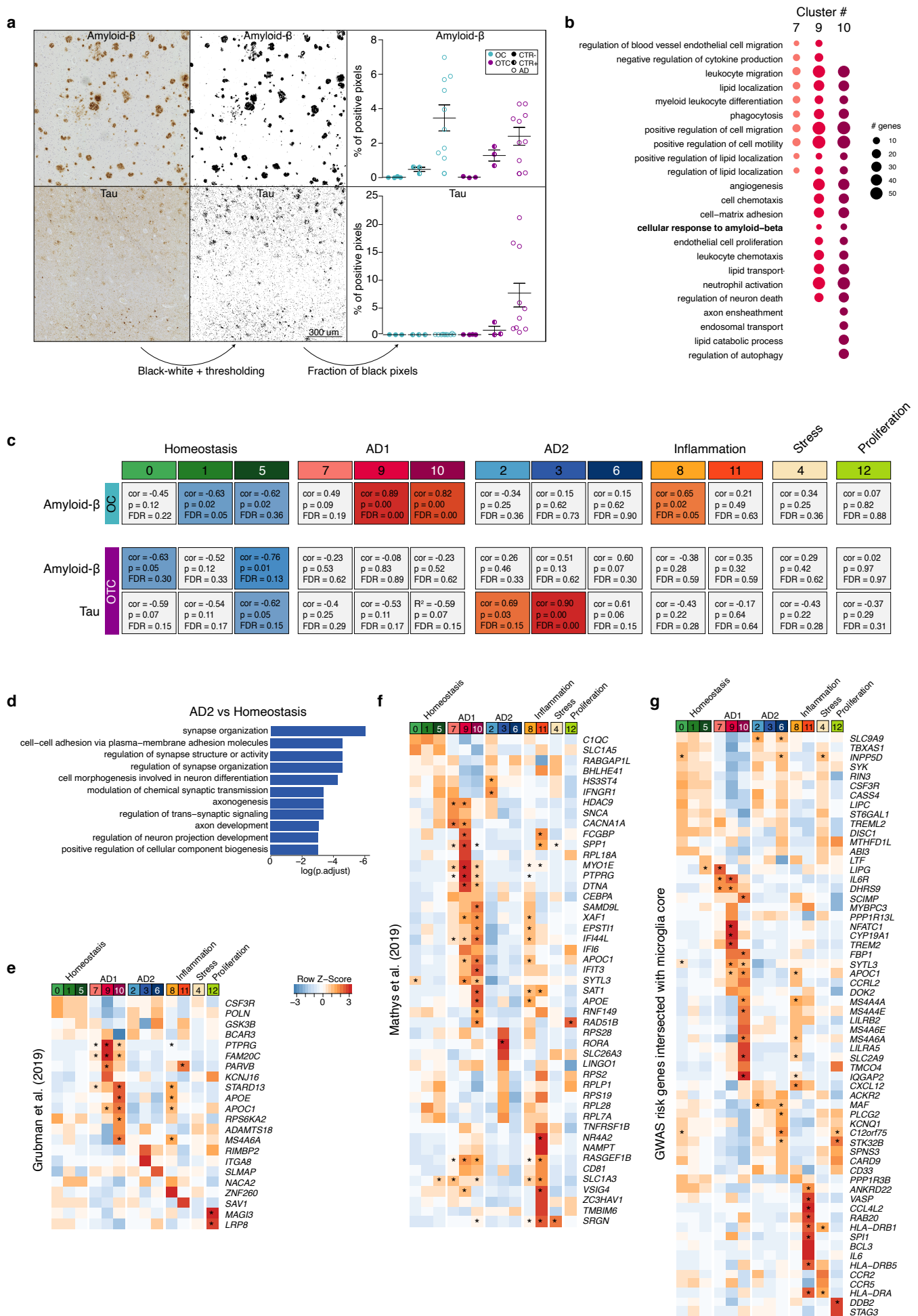

Figure S8

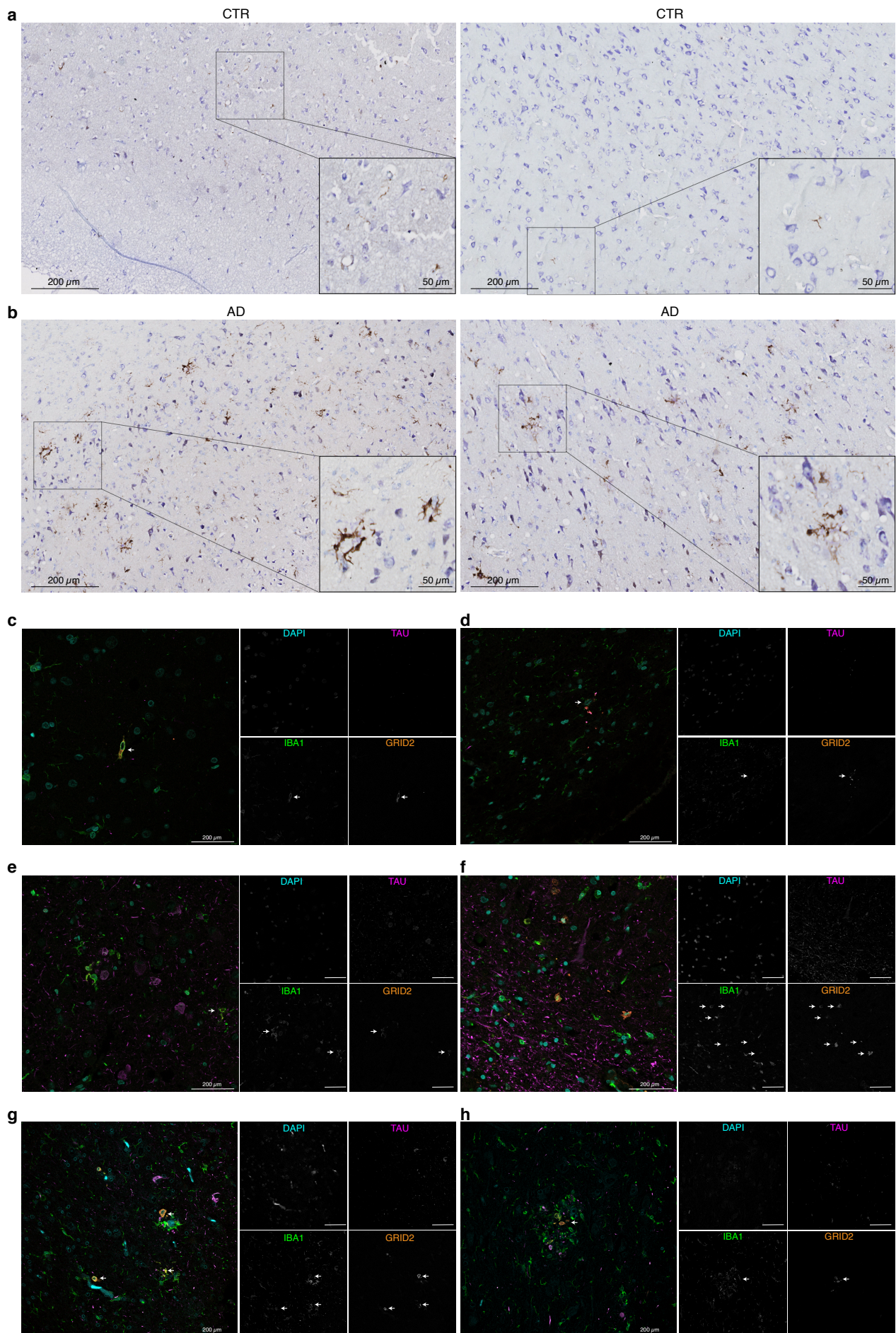

Supplement: Supplementary file 1 — Fig. S1. FACS strategy and quality control. a Nuclei were selected as DAPIpos events. Autofluorescent events were gated out with unused channels. The nuclei population was depleted for neurons and oligodendrocytes/OPCs by negative gating of NEUNpos and OLIG2pos nuclei. snRNAseq was performed on the NEUNnegOLIG2neg population. b Violin plots depicting number of expressed genes per nucleus and the percentages of expressed genes that are mitochondrial and ribosomal. c Barcode filtering strategy. X-axis depicts the number of exonic UMI counts per barcode, Y-axis the number of intronic UMI counts per barcode. Barcodes with exonic > intronic are assumed to be (partial) cells. In the analysis, barcodes that have > 250 intronic counts, > 100 exonic counts and intronic > exonic were included. d Bar plots depicting relative abundance per cell type as a fraction of the total NEUNnegOLIG2neg population per group. Circles represent individual samples. Bar indicates mean with standard error. e Distribution of cell types in the NEUNnegOLIG2neg population of each sample in bars. OC = Occipital Cortex; OTC = Occipitotemporal Cortex; CTR: non-demented controls; CTR+: non-demented controls with mild amyloid-β pathology; AD: clinical and neuropathological Alzheimer’s disease. Fig S2. Cell type marker gene expression. UMAPs depicting gene expression levels of manually selected marker genes for each of the cell types depicted in Figure 1c. Fig. S3. Bulk RNAseq of OLIG2pos and NEUNpos nuclei confirms neuronal and oligodendrocyte lineage cell type identity. a Example of a FACS plot of the nuclei sorting strategy. b Boxplots depicting transformed expression levels of the OLIG2pos samples for cell type marker genes for neurons (MAP2, RBFOX3), astrocytes (ALDH1L, AQP4, GFAP, GJA1, MFGE8), oligodendrocytes and OPCs (MOBP, PLP1, OLIG1, OLIG2, PDGFRA), microglia (CD74), pericytes (PDGFRB) and endothelial cells (CLDN5). c Principal component analysis (PCA) depicting OLIG2pos samples. Colors indic [file 401_2021_2263_MOESM1_ESM.pdf]
