# Supplementary material for: Distinct amyloid-β and tau-associated microglia profiles in Alzheimer’s disease
Source: Acta Neuropathol. 2021 Feb 20;141(5):681–96. doi: 10.1007/s00401-021-02263-w (PMC8043951; doi:10.1007/s00401-021-02263-w)
Supplement: Supplementary file 3 — Supplementary file3 (DOCX 13 KB) [file 401_2021_2263_MOESM3_ESM.docx]

**Supplemental text**

In the NEUN^neg^OLIG2^neg^ dataset, 128,764 astrocytes were obtained and 14 astrocyte subclusters were identified, which mainly segregated into two groups in the UMAP (Fig. S5a and b, Table S3), with distinct transcriptional profiles (Fig. 5c). The subclusters on the left side in the UMAP (clusters 1, 3, 7, 8, 13, 11) were enriched in expression of the well-known reactive astrocyte marker *GFAP,* whereas the other subclusters were enriched for expression of *SLC1A2, ATP1B2* and *SLC1A3 (*GLAST-1*)* which could indicate a more homeostatic profile (Fig. S5d). No AD-associated changes in subcluster distribution were identified and differential gene expression did not yield significant results correlating with amyloid-β or phospho--tau load (Fig. 1g, Fig. S5b). However, clear region-associated changes were observed between the OC and OTC samples in all donor groups (Fig. S5b). Five subclusters were significantly more abundant in OTC samples (clusters 0, 4, 6, 10 and 12) and two subclusters were significantly more abundant in OC samples (clusters 2 and 5) (Fig. S5b and e).

In AD donors, the OC samples contained only amyloid-β pathology and OTC samples contained both amyloid-β and tau pathology. To further identify whether tau-associated changes were present in AD donors, differential gene expression analysis was performed between the enriched subclusters of the brain regions for CTR(+) and AD separately, and log fold changes between the two groups were compared (Fig. S5f, Table S4). In both donor groups, the same regional effects were observed, which correlated significantly with each other (Pearson correlation = 0.84, p-value < 0.001), confirming that tau-pathology did not account for the regionally diverse astrocyte profiles in the AD donors. Recently, a disease-associated astrocyte signature in an amyloid-mouse model was identified [12]. DEGs derived from this study were not enriched in any of the astrocyte subclusters, indicating that mouse and human astrocytes are differently affected in AD. So, despite the power of the dataset to determine regional changes in astrocyte transcriptomes, AD-associated changes were not identified in this astrocyte population.
